# A Middle-Aged Man With Diplopia, Headache, and Hyperglycemia

**DOI:** 10.7759/cureus.70057

**Published:** 2024-09-23

**Authors:** Amna Noor, Amina Hafeez, Noman Safdar, Mah Rukh Nisar, Qasim Bashir

**Affiliations:** 1 Neurology, Services Hospital Lahore, Lahore, PAK; 2 Anesthesia, Services Hospital Lahore, Lahore, PAK; 3 Internal Medicine, Services Hospital Lahore, Lahore, PAK; 4 Neurology/Medicine, Services Hospital Lahore, Lahore, PAK; 5 Clinical &amp; Interventional Neurology, CMH Lahore Medical College and Institute of Dentistry, Lahore, PAK

**Keywords:** bilateral internuclear ophthalmoplegia, diabetes mellitus, diplopia, neuroplasticity, vertigo

## Abstract

A 60-year-old man with a history of diabetes mellitus and hypertension presented with diplopia and vertigo for two days. These symptoms had a gradual onset, were progressive, and were associated with a headache that had been present for 15 days. His blood sugar level was 376 mg/dl. His convergence and ability to adduct when looking laterally were affected bilaterally. Additionally, he presented with right exotropia on primary gaze and no ptosis. Brain imaging was normal. Following the rest of the work-up, diabetes mellitus was independently established as the cause of bilateral internuclear ophthalmoplegia in this patient. High blood sugar affects the brain at the molecular level by causing vascular damage, oxidative stress, and decreasing neuroplasticity.

## Introduction

A long-term follow-up study of 65 patients with internuclear ophthalmoplegia (INO) showed that bilateral INO has a frequency of 33.8% [1]. Various conditions have been known to cause INO, with multiple sclerosis being among the most common ones and associated with early presentation of the disease [2]. Over the last few years, cases of wall-eyed bilateral internuclear ophthalmoplegia (WEBINO) have connected with conditions previously not known to cause it. In patients with parkinsonism and supranuclear palsy, WEBINO has been observed over time, as reported by Matsumoto et al. [3]. In addition, a woman with systemic lupus erythematosus developed bilateral INO after COVID-19, and a variant was seen with unilateral infarction of the pons [4,5]. Therefore, it can occur due to neurodegeneration, brainstem stroke, autoimmune phenomena, demyelination, and infections.

## Case presentation

**Figure 1:**
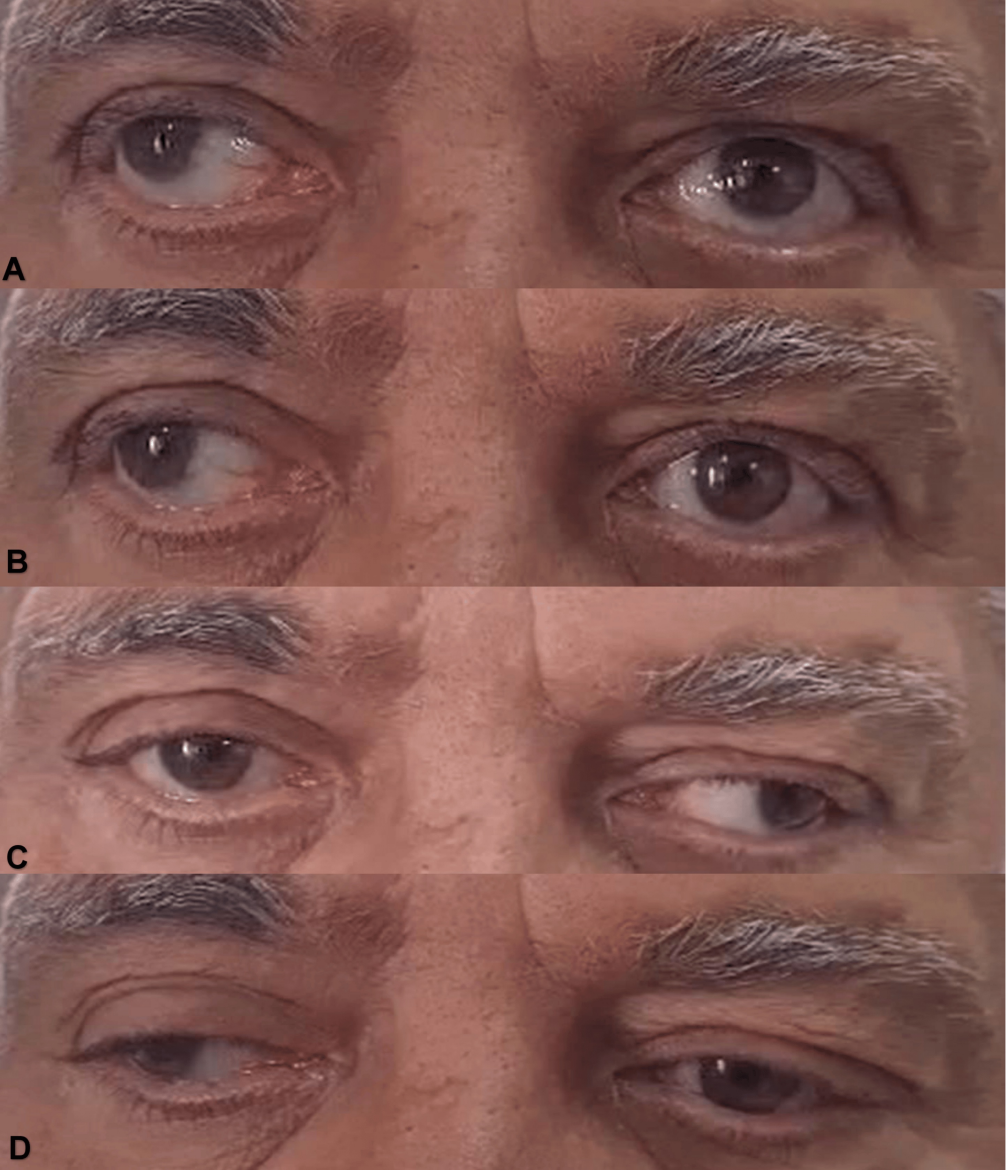
Eye movements in different gaze positions. (A) Upward movement, (B) right abduction, (C) left abduction, and (D) downward movement.

A 60-year-old man with a 25-year history of diabetes mellitus (DM) and a 10-year history of hypertension presented with diplopia and vertigo for two days. These symptoms had a gradual onset and were progressive in nature, associated with a headache that had been present for 15 days. He had no history of previous neurological deficits, fever, weight loss, vomiting, alcohol use, recreational drug intake, memory loss, cognitive deficits, traumatic brain injury, or neurosurgical intervention. Upon clinical examination, the patient was oriented in time, place, and person; he was afebrile and vitally stable. However, his blood sugar level was 376 mg/dl. His convergence and ability to adduct when looking laterally were affected bilaterally. Additionally, he presented with right exotropia on primary gaze and no ptosis (Figure 1).

**Figure 2:**
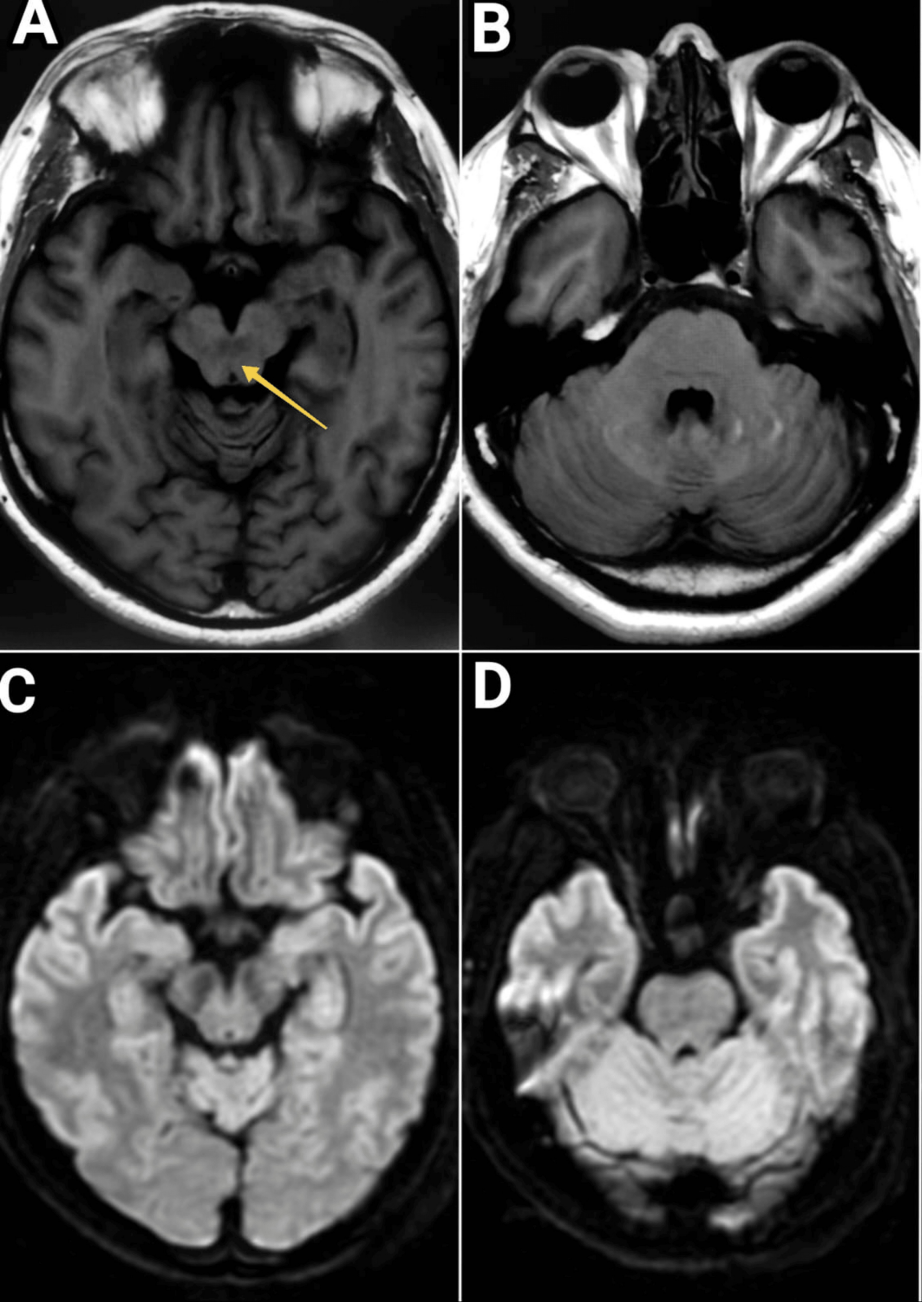
MRI brain shows a lesion-free MLF in the midbrain and pons. (A) T2 FLAIR at the level of midbrain, (B) T2 FLAIR at the level of pons, (C) DWI at the level of mid brain, and (D) DWI at the level of pons. FLAIR: fluid-attenuated inverse recovery; DWI: diffusion-weighted imaging; MLF: medial longitudinal fasciculus.

Following the rest of the neurological examination, visual acuity, color vision, visual fields, and direct and indirect light reflexes were all normal. An examination of the fundus with a slit lamp showed a bilaterally normal optic disc and macula. There were no signs suggesting cerebellar deficit, and the rest of the cranial nerve examination was completely fine. Infectious conditions were ruled out due to the absence of fever symptoms and signs of neck rigidity. Cerebrospinal fluid (CSF) analysis showed glucose to be 75 mg/dl, protein 35 mg/dl, and white cell count of zero per mm^3^. The patient's HbA1c value was 10.3%, indicating poor control of blood sugar levels in the past months. Other laboratory parameters were normal. The chest X-ray and abdominal ultrasound were normal. CT and MRI brain scans showed normal results on T1, T2, diffusion-weighted, and fluid-attenuated inverse recovery sequences (Figure 2). Demyelination was ruled out based on clinical history and normal MRI. Magnetic resonance angiography brain was also normal. Bilateral carotid Doppler showed 30% stenosis of the right and left internal carotid artery. Echocardiography revealed a 60% ejection fraction with normal biventricular systolic function, showing no source of embolization. Midbrain and pontine infarction were ruled out based on the combined work-up and examination. MRI cervical spine showed age-related degenerative changes with neural compromise. A repetitive nerve conduction study of the bilateral ulnar, facial, and accessory nerves was performed and gave a normal response. In addition, electromyography showed no abnormalities. Antinuclear antibody (ANA), rheumatoid factor (RF), and anti-double-stranded DNA (anti-dsDNA) antibody were found to be negative. On given history, examination, and laboratory results, any possible autoimmune phenomenon was also ruled out. The patient was diagnosed with bilateral INO. Based on the history and extensive work-up, most known or described causes of bilateral INO were ruled out. The patient was prescribed cinnarizine and beta-histidine to control vertigo symptomatically. He was also advised to use an eye patch alternatively on both eyes during the daytime. Gait training also helped the patient mobilize early. After strict blood sugar control for three months, the symptoms gradually reverted. It was established that DM was the sole cause of bilateral INO in this patient.

## Discussion

**Figure 3:**
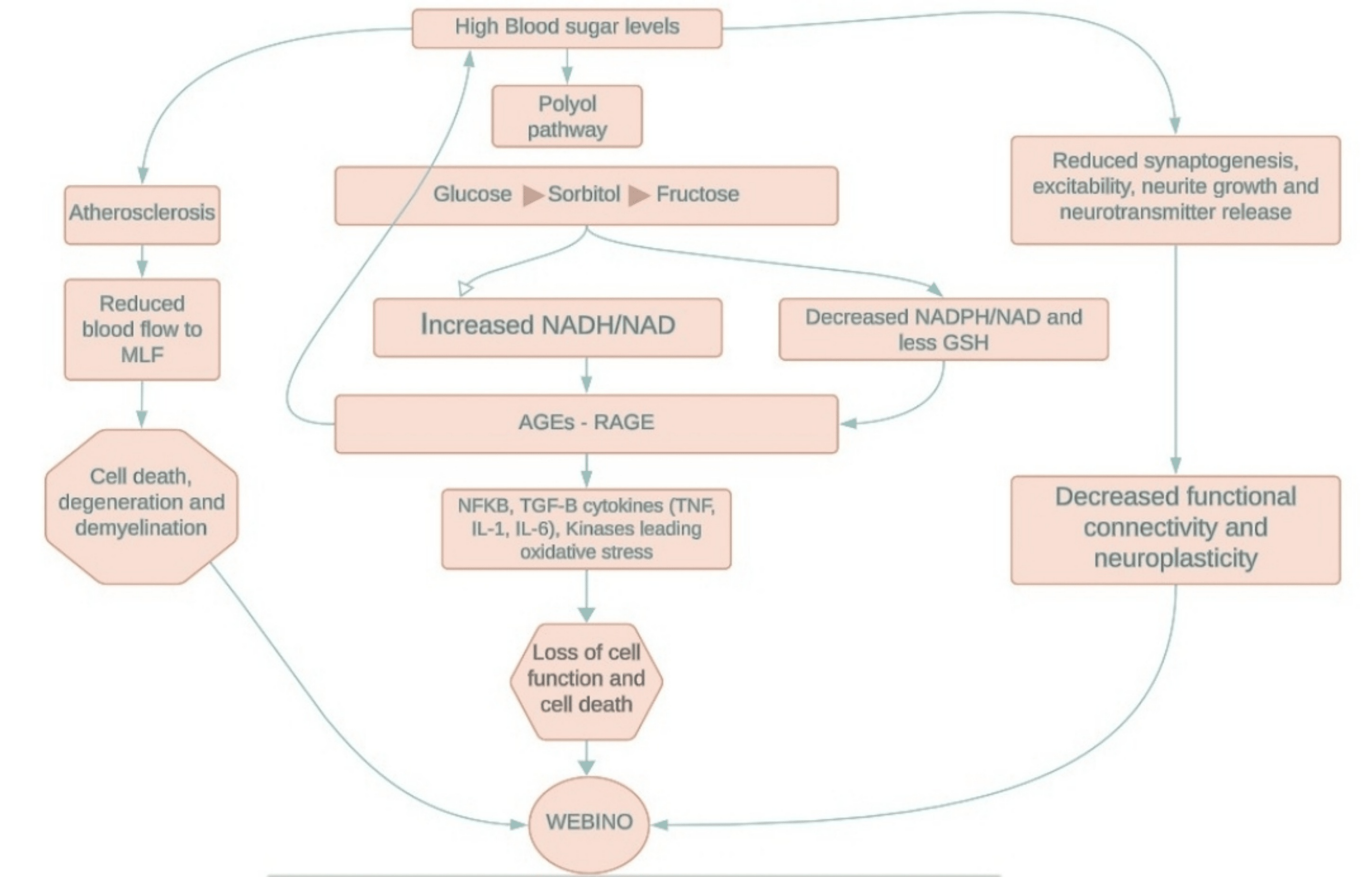
Pathogenesis of INO. (1) Ischemia can lead to decreased blood flow to MLF. (2) An increase in fructose production through the polyol pathway leads to an increased ratio of nicotinamide adenine nucleotide + hydrogen to oxidized form (NADH/NAD) and a decreased ratio of reduced nicotinamide adenine dinucleotide phosphate + hydrogen to oxidized form (NADPH/NADP) and less glutathione in reduced form; therefore, oxidative stress caused by the increased production of AGEs and their interaction with receptors (RAGEs) leads to the formation of mediators that cause loss of cell function and death. (3) Loss of neuroplasticity. AGEs: advanced glycation end products; INO: internuclear ophthalmoplegia; MLF: medial longitudinal fasciculus; NF-κB: nuclear factor kappa B; RAGEs: receptor for AGEs; TGF-β: transforming growth factor beta; TNF: tumor necrosis factor; IL: interleukin; WEBINO: wall-eyed bilateral internuclear ophthalmoplegia. Image credit: Amna Noor.

The medial longitudinal fasciculus (MLF) is a paired structure of highly myelinated nerve fibers present nearly at the midline of the brainstem and is responsible for conjugating and synchronizing eye movements. The MLF transmits adduction signals from the abducens nucleus to the medial rectus subnucleus of the oculomotor nerve [2]. It is rare to find high blood sugar levels presenting only with the inability to adduct bilaterally and associated horizontal nystagmus in the opposite eye, leading to diplopia and vertigo as presenting symptoms. Affecting a structure like MLF without an apparent structural lesion on MRI was found to be of interest. It led to the establishment of DM as one of the causes of bilateral INO at the molecular level. While there is not a direct link between diabetes and the MLF, diabetes-related complications such as vascular damage, generation of reactive oxygen species, and destruction of functional connectivity can affect the MLF indirectly, as summarized in Figure 3. Diabetes is associated with vascular complications such as atherosclerosis and microvascular disease.

MLF in the dorsal tegmentum of the midbrain is supplied by the P2 segment of small perforating arteries arising from a posterior cerebral artery. In the pons, it is supplied by perforating branches of the basilar artery [6]. Vascular changes can affect blood flow to MLF. Reduced blood flow to MLF can result in cell death, degeneration, or demyelination, compromising the function of MLF and neural pathways involved in conjugate horizontal eye movement.

High blood sugar levels increase fructose production in the brain through the polyol pathway. This results in decreased availability of a reduced form of glutathione to combat oxidative stress. Moreover, it increases the production of advanced glycation end products (AGEs), which interact with AGE receptors (RAGEs), leading to the formation of mediators of oxidative stress in the brain. This, in turn, leads to inflammation and loss of cell function [7].

Although the brain depends on sugar as its fuel for energy, Vera Novak proposed that high blood sugar levels can affect the brain’s functional connectivity, which connects the brain regions that share functional properties [8]. The ability of the brain to adapt to extrinsic or intrinsic stimuli by reorganizing its functions, structure, and interconnections is referred to as neuroplasticity [9]. Metabolic derangements such as high blood sugar levels, obesity, poor sleep, and a sedentary lifestyle have already been known to impact neuroplasticity negatively [10]. In his review, Hotspots for the Use of Intranasal Insulin, Shpakov et al. discussed possible neuroprotective mechanisms of intranasal insulin (INI) administration through various signaling cascades in neurons and glial. Updated in April 2023, INI results in neurogenesis, axonal growth, angiogenesis, reduction of inflammation, decreased cell death, and improvement in neuroplasticity [11]. Studies involving the use of insulin and controlling high blood sugar levels in reverting and preventing adverse effects, such as memory loss and cognitive decline in people with diabetes, can lead to finding a new avenue for the treatment of complications of diabetes in the brain.

## Conclusions

Diabetes can affect our body at multiple levels. Its influence on the brain at both cellular and molecular levels can directly result in neurological deficits. Bilateral INO is one of those conditions that can be caused by high blood sugar levels without any changes in imaging studies. We proposed three probable mechanisms that can contribute to WEBINO. The pathogenesis involves effects on neuroplasticity, which leads to decreased functional connectivity. It also results in the production of AGEs at the molecular level, resulting in oxidative stress at the cellular level. Lastly, reduced blood flow due to atherosclerosis causes cellular demyelination and cell death.
